# Microservice Security Framework for IoT by Mimic Defense Mechanism

**DOI:** 10.3390/s22062418

**Published:** 2022-03-21

**Authors:** Fei Ying, Shengjie Zhao, Hao Deng

**Affiliations:** 1 College of Electronic and Information Engineering, Tongji University, Shanghai 201804, China; 23yingfei@tongji.edu.cn; 2 Key Laboratory of Embedded System and Service Computing, School of Software Engineering, Tongji University, Shanghai 201804, China; 3 School of Software Engineering, Tongji University, Shanghai 201804, China; denghao1984@tongji.edu.cn

**Keywords:** mimic defense, container-based cloud, mimic transformation

## Abstract

Containers and microservices have become the most popular method for hosting IoT applications in cloud servers. However, one major security issue of this method is that if a container image contains software with security vulnerabilities, the associated microservices also become vulnerable at run-time. Existing works attempted to reduce this risk with vulnerability-scanning tools. They, however, demand an up-to-date database and may not work with unpublished vulnerabilities. In this paper, we propose a novel system to strengthen container security from unknown attack using the mimic defense framework. Specifically, we constructed a resource pool with variant images and observe the inconsistency in execution results, from which we can identify potential vulnerabilities. To avoid continuous attack, we created a graph-based scheduling strategy to maximize the randomness and heterogeneity of the images used to replace the current images. We implemented a prototype using Kubernetes. Experimental results show that our framework makes hackers have to send 54.9% more random requests to complete the attack and increases the defence success rate by around 8.16% over the baseline framework to avoid the continuous unknown attacks.

## 1. Introduction

Nowadays, wireless sensor networks (WSN) are developing rapidly with the support of the artificial-intelligence-enpowered Internet of things (AIoT), as cloud computing and AI can help WSNs overcome the challenges of real-time end-to-end data processing. Currently, microservices and containers have become the de facto approach for deploying IoT applications in cloud. The microservice model divides an application into a set of loosely coupled and collaborative components. These components then can be efficiently encapsulated and deployed using lightweight container-based virtualization. Thanks to its high flexibility, portability, and scalability, this architecture has been widely applied in scenarios such as IoTs, smart vehicles, and fog/edge computing [1,2,3].

Despite its advantages, container-based virtualization possesses a major drawback: if a container image (We used image, container image, and Docker image inter-changeably in this article) contains security vulnerabilities, all mircoservice components related to this image are in a high risk state [4]. Malicious parties now frequently exploit vulnerable images to gain control of the entire microservice architecture [5]. Moreover, with the continuous expansion of the IoT in scale, a large number of heterogeneous sensor devices connected to the IoT for forwarding and processing huge data. This make unknown vulnerabilities in microservices a potential security threat to sensor networks.

Therefore, it is crucial to address the container image security issue with the utmost urgency. State-of-the-art solutions [6,7] attempts to suppress this issue by applying vulnerability scanning tools on container images at build time or run-time. However, this approach demands an up-to-date vulnerability database, which is not available all the time. Moreover, this approach is ineffective to undisclosed vulnerabilities.

In this paper, we propose a mimic-defense-based framework for container image security, which can actively defend against unknown vulnerabilities from continuous attack without a vulnerability database. The core idea is to construct a mimic image pool, where the images’ variants are supposed to execute the same operations and output the same results. If any inconsistency is detected, we can then infer that there exists a security threat in images. Afterwards, the mimic defense platform will select another equivalent executor (e.g., image variant) from the heterogeneous resource pool to replace the current image variants to prevent future attack. Mimic defense method can be a supplement to a traditional static defense method; it is able to solve uncertain threats based on unknown vulnerabilities, and backdoors in different fields of cyberspace, in order to protect sensor-generated data and AI calculations.

Note that how we select the replacement image can be crucial to system security. Container images are composed of multiple read-only layers; the base layer merely contains a minimal operating system. We can add software components to the image by creating an overlay. We can formulate the relationship of image replacement as a mimic image graph. Each node in the graph represents a set of image variants. If there are differences between the two image groups due to the different levels of the images, there will be edges between nodes. Due to the fact that the greater the distance between two nodes, the more difficult it is to break through one image variant group to the other. The optimal migration strategy can be obtained by maximizing the randomness and migration distance, enabling the mimic defense framework to have the optimal active defense power. In practice, our framework can protect large-scale IoT services for governments and militaries (e.g., commands to operate the hydropower station), and helps big companies (e.g., Amazon AWS or Azure Cloud) to protect their most sensitive AI services. The contribution of this paper can be summarized as follows:

1. We propose a novel container image security framework to proactively defend against continuous attacks from unknown vulnerabilities;
2. We propose a novel graph structure to maximize the randomness and heterogeneity for the image group transformation, which enhances the ability of our framework to resist continuous unknown attacks;
3. A prototype is developed using Kubernetes and the experimental results show that our framework can achieve a better active defense power to defend against random attacks, and increases the defence success rate by around 8.16% over the baseline framework to avoid the continuous unknown attacks.

## 2. Related Works

In this section, we introduce the containerized technology in Internet of Things (IoT), existing methods for microservice security defense, and active security defense method, which are closely related to our work.

### 2.1. Containerized Technology in IoT

As a core of Industry 4.0, the IoT refers to a wide variety of physical devices connected via the Internet. IoT devices can exchange information and complete task without human assistance. IoT may generate massive amounts of data that have to be processed and stored, which leads to the concept of layered architecture. The “microservice architecture” has emerged as a popular aspect as it enables the application to be developed through a bunch of loosely coupled lightweight services, which increases the scalability of deployment. Recently, many existing works have studied the deployment of “microservice architecture” in IoT scenarios. In [8], the authors combined a single-board computer (SBC) running Docker with a microcontroller to create an automated guided vehicle. Ref. [9] proposed a smart building architecture in which sensors share data with SBC running microservices. Ref. [10] used Docker to create a Gateway for IoT and customize the IoT platform. In addition, Ref. [8] compared different virtualization methods with Cloud Computing (CC) dominated by virtual-machines (VM), and found that Docker presents minimal overhead in terms of CPU, memory, storage and network performance. Ref. [11] also analysed Docker in terms of fault tolerance of services and service deployment.

Existing works show that it is feasible to deploy Docker containers in IoT devices. Many architectures were presented but few of them consider the security issues. Default Docker installation does not verify an image authenticity. When a user interacts with an image for the first time through a Docker command, trust is automatically established with the image publisher. Previously, Yao et al. [12] studied building trust in selected gaming virtual communities. Ref. [13] proposed a blockchain-based decentralized docker trust (DDT) solution to avoid the Denial-of-Service (DoS) attacks.

### 2.2. Microservice Security Defense Methods

By virtualizing physical servers and storage devices, a cloud environment can reduce resource-sharing costs and provide multiple replicas to users around the clock from all over the world. The greatest disadvantages of cloud systems are security- and privacy-related, which are much more serious than traditional systems [14]. Influenced by COVID-19, cloud enterprise solutions are becoming more widely used in education, healthcare, e-commerce and supply chain industries. As a result, there are more cyber security attacks leading to healthcare emergencies in this period [15].

Container-based solutions have become popular in developing cloud applications [16]. Security concerns exist irrespective of the IT industry, education, banking sector based on recent perspectives. Recently, Docker, and container technology in general, has become increasingly popular and offers significant benefits to developers and companies, including the efficiency, cost savings, consistency, reliability, and scalability of the entire development process. However, for running large-scale sensitive applications, ensuring the security of container-based solutions is still a big challenge [17]. Multiple security risks exist when using Docker to orchestrate the cloud environment, which include internally deployed malicious applications, infected containers running on the cloud, and malevolence or semihonest hosts [18]. However, among these risks, most container-based cloud attacks are aimed at attacking container images [19]. Nowadays, more than 30% of official Docker images contain high-severity vulnerabilities [20]. Therefore, protecting container images from vulnerabilities is the core of container-based cloud security.

With the help of a risk library, there are many methods proposed to deal with image-security issues on container-based clouds. The most popular methods take uses of Linux features (i.e., CGroup and Capabilities) to isolate the hardware resource, and to divide the privileges, respectively [21]. They provide the general security protection of the storage and networking for containers. Brady et al. [22] use a continuous integration and continuous deployment (CI/CD) system to protect the security of Docker images. It scans the image when it is created, updated, and deployed, so the image is reliable throughout its life cycle. Kritikos et al. [23] present a configuration metamodel to recognize all the vulnerable risk from the mate-data. It performs vulnerability detection on each layer of the image, and forms a risk analysis report by mapping the detected result to the corresponding risk level. However, these methods still suffer from effectiveness problem. They have to scan all container images at build time or running time by vulnerability detection tools. But there is no guarantee that the virus database is up-to-date and that they can grasp all attacking viruses.

### 2.3. Active Security Defense Method

Different from the traditional defense methods, active defense methods are used to protect the system from several large-scale, complex, and covert attack behaviors [24,25,26]. The existing works can mainly be classified into three categorises: dynamic intrusion-detection methods [27], machine-learning-based methods [28], and cyber mimic-defense methods [29]. The dynamic intrusion-detection method was first proposed in the framework of moving-target defense (MTD), which increases the complexity of the attacker by dynamically moving the attack surface of the protected system [30]. Based on this technology, Alavizadeh et al. [31] further proposed the diversity MTD technology, which improves cloud security by deploying different operating systems into various variants. Bardas, et al. [32] introduces the MTD platform to increase the difficulty of attacks by allowing any running component of the IT system to be replaced with the original version.

**Figure 1:**
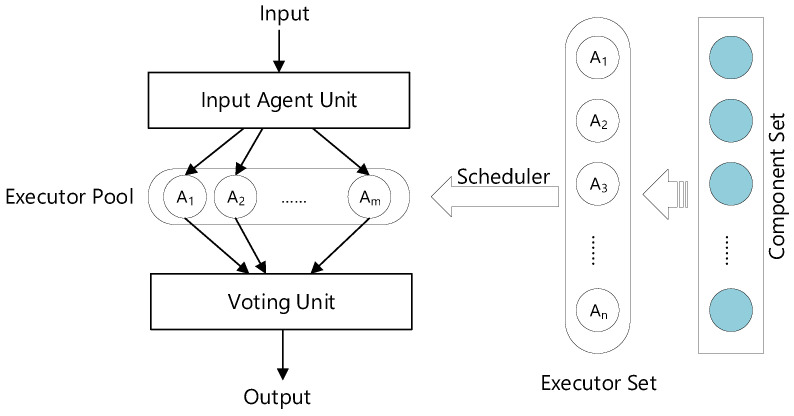
The dynamic heterogeneous redundancy architecture.

In addition, to detect attack activities before encountering an attack, a cyber mimic-defense framework (CMD) is proposed [33] based on dynamic heterogeneous redundancy (DHR) infrastructure [29]. As shown in Figure 1, the CMD framework mainly consists of an input agent unit, executor pool, and voting unit. The executor pool contains a set of equivalent variants which are dynamic heterogeneous redundancy and are functionally equivalent. By receiving the input request from the agent unit, the voting unit generates a relatively correct unique output by voting on the executor pool. The security of the mimic defense framework depends on the heterogeneity of the executor pool and the randomness of the selection scheduler of the executor set. The heterogeneous executor pool is reconstructed when the vulnerabilities are triggered. In this way, the attacking cost and difficulties of the microservice based on mimic defense framework can be increased [34]. Nowadays, there are several works based on this framework to protect the system’s security, such as the mimic-defending web server [35], or the mimic defense construction router [36]. These implementations prove that the mimic defense framework is effective in defending against unknown vulnerabilities.

In the DHR architecture, the executor pool is consists of functionally equivalent software or hardware developed independently by different teams with different technical backgrounds. Therefore, there is an extremely low probability of consistency vulnerability among them. For the attackers, even if they control a part of the executors, it is not guaranteed that all the executors can respond to vulnerability attacks consistently [29]. Therefore, the attacks based on unknown vulnerabilities will lead to inconsistencies in executors’ responses in some dimensions (e.g., API response, disk I/O, database operation, etc.), which can be identified by the mimic defense mechanism. As a result, it fills in the blind spots of traditional defense.

## 3. Mimic-Defense-Based Microservice Security Framework

This section presents the detailed design of the mimic-defense-based microservice security framework, named MDSF.

### 3.1. MDSF Overall Architecture

As discussed earlier, IoT services are facing several security and privacy challenges. To protect the microservice against the backdoor attacks, we propose a mimic defense framework and use Kubernetes to manage the container and the images for the microservice. The framework is expected to protect the authorization of IoT devices and their data from malicious attacks. For example, if certain manufacturing data is not expected to be collected at certain times or places, the framework should be able to distinguish between allowed and disallowed actions related to the specific context in which the action occurs. In the design of MDSF, a main component is to take advantage of redundancy for the safety improvement. This main part is called the Mimic Defender, which forwards the requests to several equivalent executors. Therefore, if any equivalent executor outputs a result different to others, we can identify an attack.

**Figure 2:**
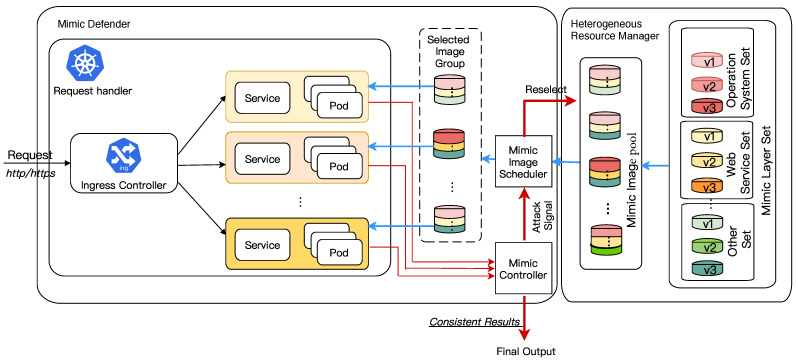
MDSF overall architecture.

Considering that IoT services are moving from the cloud to the edge because of security, latency, autonomy, and cost, running IoT services creates a new management challenge for system administrators and developers. Kubernetes provides a common platform that could be used for deploying IoT services at the edge. Therefore, we leverage the concepts of Kubernetes to build our microservice security framework based on mimic defense. The general structure of the proposed MDSF is illustrated in Figure 2. Further details on MDSF and its supportive components are given in the subsequent sections.

### 3.2. Heterogeneous Resource Manager

The heterogeneous resource manager (HRM) is the heart of the MDSF. It performs the core function of generating multiple redundant virtual images as equivalent executors. With a set of equivalent actuators performing the same operation, inconsistent outputs can detect attacks due to image vulnerabilities, such as data shadowing, blocking access to data, granting access to data, revoking permissions, installation control, saving state, and disabling intents [37]. HRM consists of several components which are introduced as follows.

#### 3.2.1. Mimic Layer Set

This component manages a set of layers correspond to certain instructions in Docker image’s Dockerfile. The instructions are for the components to build the image, such as the operation system, mounted applications (e.g., Apache), etc. It is of note that there exists equivalent candidates for each component. For example, Centos, Ubuntu, and Fedora are equivalent candidates for building the operating system. Every layer of the Docker image can be substituted with an equivalent candidate. In this way, we can build different docker images with equivalent function through the Dockerfile based on the mimic layers. For example, the mimic layer set may contain several OS candidates (e.g., Ubuntu, CentOS, Fedora). We can choose one from the mimic layer set, and write it in the Dockerfile to build the image.

#### 3.2.2. Mimic Image Pool

Images are templates from which containers are launched. We transparently overlay these layers to create an Docker image. The mimic image pool consists of all the images which are built from Dockerfiles with the same number of layers and each layer is selected from the mimic layer set. The layered architecture have a couple advantages. First, they are immutable. Once created, these layers are read-only which will never be changed. Second, this immutability allows images to safely build and fork off of each other. This facilitates us to organize the candidates from different layers to build an image.

### 3.3. Mimic Defender

Mimic defender is the main part of MDSF, which encompasses the essential functions to protect the system’s security and the user’s privacy. In order to fulfil its purpose, this component needs to proactively defend against attacks and can be seamlessly transplanted to the existing container-based cloud platform. Typically, the mimic defender mainly consists of the following components.

#### 3.3.1. Request handler

The request handler is responsible for exposing the service and managing external access to IoT services running in the container. We use the Ingress Controller of Kubernetes to expose http/https routes of the running services. In this way, the outside of the Kubernetes cluster can access the services running in the container through the APIs [38]. Typically, Ingress Controller plays the role of input agent unit, which replicates and distributes the same requests to the equivalent services and their corresponding pods. Pods can be analogized as machine instances to a container. Each pod is allocated its own internal IP address, and containers within pods can share their local storage and networking. A Kubernetes Service acts as a layer above the pods, which enables network access to a set of pods. Thus, we can regard these services as a mimic executor pool in DHR architecture.

#### 3.3.2. Mimic Controller

The mimic controller is used to produce the output of the system and provide the safety to the scheduler. Particularly, the mimic feedback module is responsible for comparing the execution results from several equivalent services. The consistent execution result from majority will be the final response of the system. Note that if there is not enough execution results for decision making, the mimic feedback module will ask the Ingress Controller to reconstruct the equivalent services. Most importantly, mimic feedback module will detect the inconsistent results and report to the scheduler. The inconsistent results indicate a vulnerability in the image. Meanwhile, the system may check whether all the services created from the selected image is working in an abnormal state. Otherwise, the redundancy between services is used to discover which image exists the vulnerability. In this way, IoT microservices can work in safe mode even if the base image is vulnerable.

#### 3.3.3. Mimic Image Scheduler

In MDSF, we propose a novel mimic image scheduler to avoid continuous attack. This is because the service is launched from the selected image: if we replace the vulnerable image with one not of great differentiation, the attack will easily continue. Therefore, once receiving an attack signal, the mimic image scheduler will ask the mimic image manager to reselect the image groups, following the MDSF scheduling strategy described in Section 3.5.

### 3.4. The Workflow of MDSF

**Figure 3:**
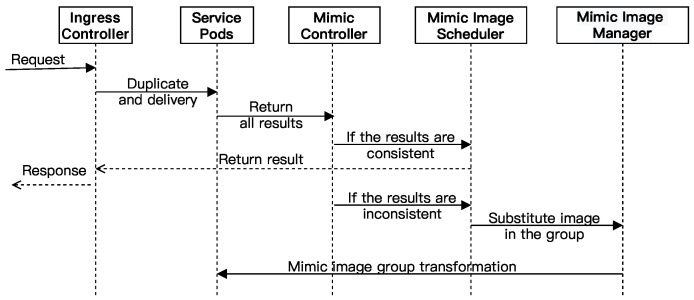
The diagram of the MDSF working process.

The diagram of the MDSF working process is shown in Figure 3. In the beginning, Ingress Controller replicates the request and passes the request to the service pods launched from their corresponding images. As the equivalent executors, these service pods will process the received same request and send their respective operation results to the mimic controller. If these results are consistent, the mimic controller will let the Ingress Controller expose the result as a system response. Otherwise, to avoid further attack from unknown hackers, the mimic image scheduler will be triggered to replace the images which launched the services with different results.

Afterwards, the original selected image group will be switched to another candidate group. This process is called the mimic image group transformation. A mimic transformation is used to effectively change the static nature of the images on which cyber attacks rely. To achieve this, when we replace the images, the first step is to stop the container and capture the state of the stopped container. Correspondingly, a checkpoint is created for a to-be-replaced container, which includes in-memory state data of all processes running inside the container. By transferring the container checkpoint from the original in-memory state to the new host’s user-space, the mimic transformation can be achieved at a lower cost.

### 3.5. MDSF Schedule Strategy

To better improve the security performance of active defenses, we create a schedule strategy based on a novel graph structure to maximize randomness and heterogeneity for the image group replacement, which will be introduced in this section. Particularly, we first give some notations to help us formally define the studied problem. Then, we introduce the graph-based scheduling strategy.

#### 3.5.1. Notations and Definitions

**Mimic layers** are a set of layers that correspond to certain instructions in the Docker image’s Dockerfile. Each mimic layer $L_{i}$ contains $m_{i}$ candidate choices marked as $L_{i_{k}},k\in \left[1,m_{i}\right]$, for a given image, $L_{i}=L_{i_{k}},k\in \left[1,m_{i}\right]$.

**Mimic images** contain a set of Docker images with a same number of layers, and each layer is selected from mimic layers defined as above. It can be denoted as a *n*-tuple $I={\left(L_{1},L_{2},L_{3},\cdots ,L_{n}\right)}^{T}$, *n* is the number of the layers, $L_{i}$ presents the *i*-th layer of the image, and *I* describes the each layer information of an individual image. Only when two images $I_{a}$ and $I_{b}$ meets $L_{i_{a}}=L_{i_{b}},\forall i\in \left[i,n\right]$, we can say images $I_{a}$ and $I_{b}$ are same, otherwise, they are different.

Assuming each layer in the image can be substituted with any other equivalent layers, we can obtain a mimic image pool with *N* different images related to the number of image layers *n* and each layer’s candidate choice $m_{i}$ as follows.

(1) $$N=\prod_{i=1}^{n}m_{i},$$

where $m_{i}$ represents the candidate number of layer *i*. *N* is the number of mimic images can be generated from the mimic layers. So all these *N* images have the same function; therefore, they can use them to create equivalent variants in container-based cloud with the mimic defense framework.

**Figure 4:**
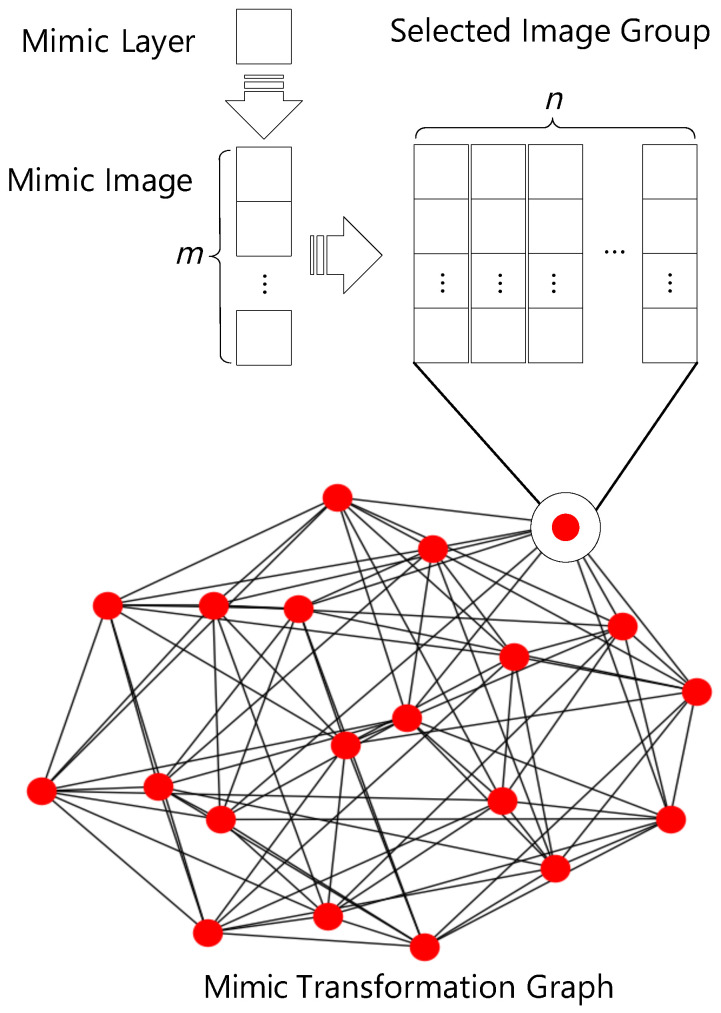
The illustration of mimic transformation graph components.

**Selected image group (SIG)** consists of a set of images selected by mimic image scheduler. The services launched from these images are functionally equivalent. If they perform differently, this suggests that at least one image has vulnerabilities which are exploited by attacker. We can denote an SIG with *n* mimic images as $SIG=\{I_{1},I_{2},\cdots ,I_{m}\}$, where *m* is the number of images in the selected image group.The total number of distinct mimic images $L(G_{a},G_{b})$ in these two groups are:

(2) $$L\left(G_{a},G_{b}\right)=rank\left(cat{\left(G_{a},G_{b}\right)}^{T}\right),$$

where $G_{a}$ and $G_{b}$ are SIG, which consist of *m* mimic images with *n* mimic layers. As shown in Figure 4, $G_{a}$ and $G_{b}$ are two matrices whose columns are the mimic layers in each image. $cat(G_{a},G_{b})$ concatenates the matrix $G_{b}$ to the right of $G_{a}$, and rank(·) is the operation of matrix rank.

The **mimic transformation graph (MTG)** represents the transformation relationship between SIGs, denoted as W=(N,E), where *N* is a set of SIG defined as above, and E⊆N×N is a set of edges, where each edge indicates the relationship between mimic images.

$MTG_{\left(n_{i},n_{j}\right)}$ indicates a mimic transformation from SIG $n_{i}$ to SIG $n_{j}$, where both $n_{i}$ and $n_{j}\in N$ contain *m* images. The distance of this transformation denoted as $D_{i,j}$ is computed by $D_{i,j}=L\left(n_{i},n_{j}\right)-m$, where $L(n_{i},n_{j})$ is the total number of distinct mimic images in $n_{i}$ and $n_{j}$ according to Equation (2). In practise, we make there exists an edge between $n_{i}$ and $n_{j}$ if and only if $D_{i,j}=1$. Thus, the edge indicates that there exists one image migration between node pairs $\left(n_{i},n_{j}\right)$. Therefore, the shortest hop between the node pairs $\left(n_{i},n_{j}\right)$ indicates the number of images should be replaced in the mimic transformation process from SIG $n_{i}$ to SIG $n_{j}$.

The process of MTG generation is described in Algorithm 1. First, we use the equivalent layers to compose the Dockerfiles (Lines 1–3). Then, we create the different images with a same function base on these Dockerfiles and push these images to the mimic image set (Line 4–6). Based on these images, we construct the graph based on the steps from Line 7 to Line 15. With the help of MTG, we can easily generate all the mimic states with present resources and all the potential mimic transformations. To better improve the security performance of active defense, we propose a schedule strategy based on our MTG, which will be introduced in the next section.
**Algorithm 1** Mimic transformation graph generation.1:**function**generateMimicImageSet(LayerNum,layers[])2:    **for** each i∈[1,LayerNum]**do**/∗Traverse mimic layer set∗/3:          Insert $layers[m_{i}]$ information into Dockerfile4:    Create image with Dockerfile and name it imageId5:    Append imageId to images[]/∗Add image to the mimic image set∗/6:    **return** images[]7:**function**createMTG(images[],m)8:    W←null; /∗Weighted graph W∗/9:    Select *m* different images from images[] as *G*10:    **while** G∉W **do**11:          Append *G* to W.Node[]12:          **for** each node in *W* **do**13:                **if** diff(*G*, node) = 1 **then**14:                     Append E(G,node) to W.Edge[]15:    **return** *W*

#### 3.5.2. Graph-Based Scheduling Strategy

In a cyberspace environment, if any vulnerabilities in the mimic layer take effect, the equivalent services will generate different results when running the application based on a mimic defense framework. The MDSF will then take mimic transformation process to protect the application, such that SIG will change to another mimic state. This process will make sure that the attacker’s experience cannot be inherited. The mimic transformation process will have good security performance when the following conditions are met:

1. The new SIG should be far away from the original SIG (heterogeneity).
2. Each image in SIG should be selected with equal probability from a mimic image pool (randomness).

We define the output of the transformation scheduling as a sequence *S*. Each node *n* selected from the MTG is added to the sequence *S* in chronological order. Accordingly, we can measure the heterogeneity of the scheduling strategy by computing the shortest number of hops between every two adjacent elements in *S* as follows.

(3) $$M\left(S\right)=min\left(\mathrm{hop}\left(n_{i},n_{i+1}\right)\right),\;i\in \left[1,n-1\right]$$

where $n_{i},n_{i+1}$ are two adjacent SIG in *S*, and $hop(n_{i},n_{i+1})$ indicates the hop number between $n_{i}$ and $n_{i+1}$ based on MTG. A larger M(S) represents a higher heterogeneity of the scheduling strategy, and the less the attacker’s experience can be inherited.

We measure the randomness of the scheduling strategy by calculating the total entropy of each element in the sequence *S* as follows.

(4) $$H\left(S\right)=-\sum_{i}p\left(n_{i}\right)log\left(p\left(n_{i}\right)\right),$$

where $p(n_{i})$ is the probability of $n_{i}$ in *S*. A higher H(S) means more uncertainty for attackers to implement the attack. To protect the application deployed on the container-based cloud, the scheduling strategy *S* should jointly consider M(S) and H(S) parts. To evaluate the security level of the scheduling strategy *S*, we calculate a weighted sum of M(S) and H(S) as follows.

(5) SL(S)=αM(S)+βH(S),

where α and β are weight parameters determined by specific scenarios. Thus, the objective of the scheduling strategy is to find a mimic image replacement sequence *S* that can maximize the SL(S).

Typically, the M(S) part is maximized when $p\left(n_{i}\right)=\frac{1}{N}$, where *N* is the total number of SIG in MTG. This means that our strategy should select each image equally from the mimic images pool. In particular, if the length of the strategy sequence is the same as the number of nodes in the MTG, each node should only appear once in the sequence *S*. Meanwhile, we define a Transformation Hops Matrix (THM) to restore the shortest distance from any two nodes in the SIG. For example, $n_{ij}\in \mathrm{THM}$ represents the shortest hops from $n_{i}$ to the $n_{j}$. Since all the SIG nodes total have *m* different images, the largest element in THM is *m*. Accordingly, we can maximize the H(S) part by generating a transformation sequence as described in Algorithm 2.
**Algorithm 2** Generate the mimic transformation sequence.**Require:** The Mimic Transformation Graph MTG(N,E)**Ensure:** The Mimic Transformation Sequence *S*1:Create an empty Transformation Hops Matrix *M*;2:**for***i* in *N* **do**3:      **for** *j* in *N* **do**4:            $n_{ij}\leftarrow$ The number of hops for the shortest path from $n_{i}$ to $n_{j}$5:            $n_{ij}$ appends to M.Node[];6:**for**t←m; *t*  >  0; t←t−1 **do**7:      **for** each element in *M* **do**8:            **if** element  <  *t* **then**9:                 element←0;10:            **else**11:                 element←1;12:      **if** There exists a Hamiltonian path in *M* **then**13:            **return** Hamiltonian path in sequence14:**return** Null

In Algorithm 2, we first generate THM, denoted as *M*, according to the algorithm on lines 1–5. Considering that the maximum number in the THM is *m*, we set the threshold to *m* and transform the THM according to lines 6–11. More specifically, we mark the element in THM as 1 if it is greater than *m*, and 0 otherwise. Next, we examine the Hamiltonian path in *M*. If it exists, we output it as the result (lines 12–13). Otherwise, we reduce the threshold to m−1 and repeat the steps from lines 7 to 13. Finally, if we cannot find any Hamiltonian paths when the threshold *t* drops to 0, we output a Null value. Thus, we can transform the SIG from one group of different images to another group, so as to avoid inherited attacks.

In addition, we can prove that Algorithm 2 can always find a nonempty solution to the sequence of mimic transformations. This is because that assuming the mimic image pool has *n* different images and each SIG consists of *m* different images, totally there is $N=C_{n}^{m}$ nodes in the MTG, and the number of neighbors in each hops of a given node is calculated as ${Nei}_{i}=C_{m}^{i}\times C_{n-m}^{i}$. We can easily prove that ∀m,n≤3, $\sum_{i=1}^{m}Nei_{i}>\frac{C_{n}^{m}}{2}$, according to Dirac’s theorem that was already proved [39]. This indicates that the updated *M* will always exists a Hamiltonian path, in the worst case, when the threshold drops to 1.

## 4. Implementation and Experimental Result

Here, we describe how our proposed framework was implemented. Then, we present the performance of our system in two real scenarios and analyze the experimental results.

### 4.1. Implementation

**Figure 5:**
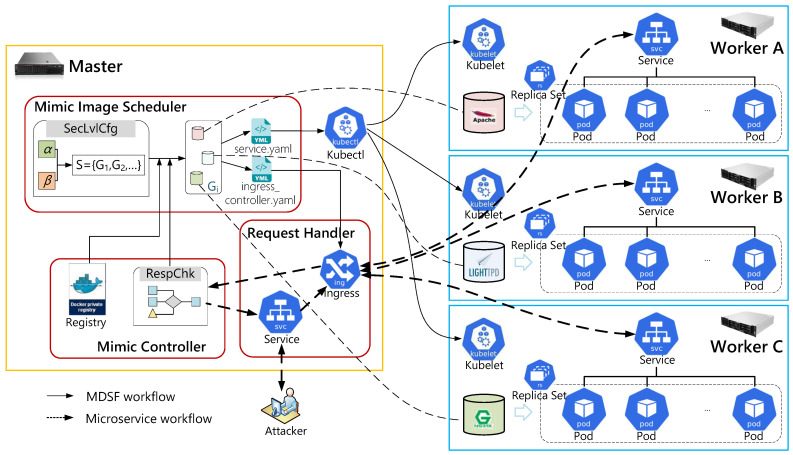
The systematic implementation of MDSF. The icons in blue are Kubernetes native components, and modules in red boxes are the developed plug-ins.

This section explains the implementation details of our framework. As shown in Figure 5, our system is developed based on the Kubernetes architecture, where the blue icons are Kubernetes native components, and modules in red boxes are the developed plug-ins. Mimic Controller and services are deployed on one master node and three worker nodes, with the configuration settings in Table 1. In this platform, each worker runs a set of logical pods launched from the SIG. These pods perform the same service to reduce the response time. Actually, the workflow of MDSF can be divided into two aspects. The execution workflow indicates the deployment detail of MDSF, and the microservice workflow presents how microservices work under MDSF deployment.

**Execution Workflow:** Initially, the security level is set by α (i.e., heterogeneity weight) and β (i.e., randomness weight) from SecLvlCfg unit in the Mimic Image Scheduler (MIS) module, which calculates the SIG sequence. Once the RespChk unit reports an exception, the MIS will reselect the images from the Docker private registry and write the selected images information to *service.yaml* and *ingress_controller.yaml*. The modified *service.yaml* will notify Kubectl, which tells each Kubelet on the worker nodes to create a service with the specified image. After the services are created, ReplicaSet on each worker node adjusts the number of pods to accommodate request pressure and reduce response time.

**Microservice Workflow:** We modify the configuration of Ingress so that the request traffic can be mirrored to the equivalent functional services on worker nodes. For example, these services are Apache, Lighttpd, and Nginx. By modifying *ingress_controller.yaml*, Ingress will expose requests from cyberspace (possibly by an attacker) and transparently forward them to services on designated worker nodes. Afterwards, all the services will individually handle the request and return the response to the RespChk unit for checking through ingress. The consistent response indicates there are no attacks against backdoors and vulnerabilities, which will be the final response of the system. Otherwise, the RespChk unit triggers the MIS to reselect a new SIG and the Execution Workflow has to be repeated.

### 4.2. Experimental Design

This section presents the experiments showing how MDSF effectively defends against continuous network attacks from the unknown vulnerabilities.

We consider the stateless service IoT microservices which are deployed on a container-based cloud with Kubernetes platform. In MDSF, the detailed information of mimic layer sets used in the experiment is shown in Table 2. Our experiment makes these sets contain publicly known zero-day vulnerabilities. For example, Nginx with version 1.10 is in the set, if the current time is the year 2016, we can treat CVE-2017-7529 as a zero-day vulnerability. When the IoT microservice is deployed using the basic image with nginx as web server, the attack will be success through CVE-2017-7529. Based on this mimic layer set, we can easily generate a mimic image set through Dockerfile. The relationship of the images in the mimic image set with the mimic layer set is shown in Table 3.

**Figure 6:**
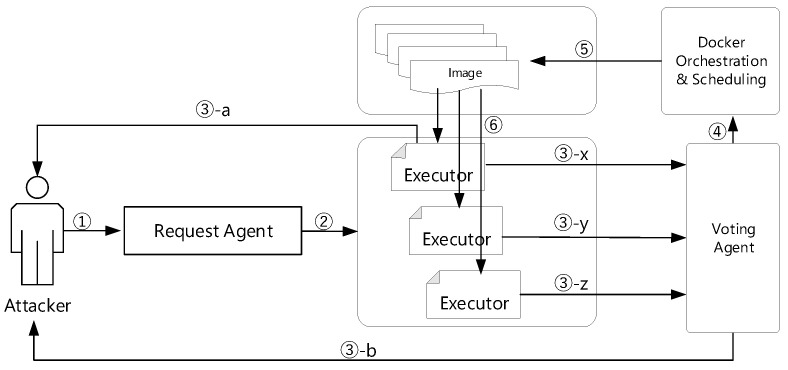
The process of hackers trying to attack the IoT microservice by sending requests.

In our experiments, we simulate hackers trying to attack the IoT microservice by sending requests. As shown in Figure 6, ➀ the attacker embeds zero-day vulnerabilities into a request. ➁ The request is transmitted transparently through the request agent, which copies and sends the request to several equivalent executors. Initially, these executors are launched from images in the selected images group. ➂ The responses of these executors are marked as ➂-x, ➂-y, ➂-z, respectively. If any of these equivalent images contain vulnerabilities, the response will be different and the response will be ➂-a, which means that the attack is successful. Otherwise, we can recognize the attack (➂-b). At this time, ➃ Docker Orchestration & Scheduling module will be triggered, ➄ the image will be replaced by modifying the yaml file in Kubernetes. As a result, ➅ the executor will synchronously update.

We conduct the experiments to evaluate the active defense ability of MDSF in two scenarios. One is to avoid the unknown attacker’s first attack on microservices at any time. (Scenario 1). Another one is to consider how to prevent continuous unknown microservice attacks from hackers (Scenario 2). Details of these two scenarios are presented below.

**Scenario** **1.** *We generate six requests to attack the mimic image set containing six images with zero-day vulnerabilities, as shown in Table 3. When an IoT microservice launched from these images receives an attack request, the microservice will be attacked by hackers. As a result, it will output wrong results and disclose private information to the outside.*

**Scenario** **2.** *To check whether a continuous attack can success, we generate the same harmful requests continuously to attack the mimic image set containing six images, one of which has a zero-day vulnerability. When an attack is detected, Mimic Image Scheduler will ask Mimic Image Manager to update the members of the Selected Image Group, which can prevent attackers from successfully attacking the microservices again.*

To evaluate the capabilities of our framework on these scenarios by taking different MDSF scheduling strategies, we set α and β in Equation (5) to different values, as shown in Table 4. In particular, $S_{1}$ and $S_{3}$ are the strategies that give priority to generating heterogeneity- and randomness-replacement sequences, respectively. $S_{2}$ is a strategy which tries to balance the heterogeneity and randomness of the generated sequence. In addition, we take the traditional moving target framework [33] as baseline, noted as $S_{0}$.

### 4.3. Experimental Results

To evaluate the security performance of different strategies under the abovementioned two scenarios, we report the experiment results in this section.

#### 4.3.1. Study of the Randomness Attack

**Figure 7:**
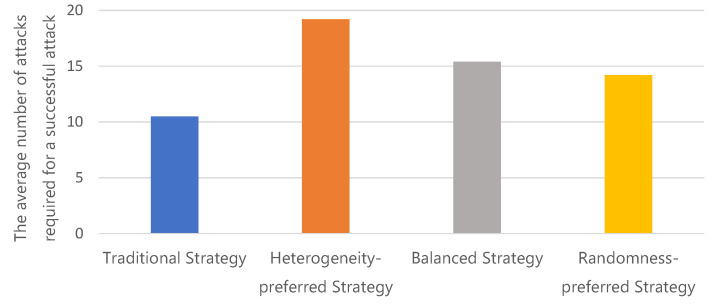
The average number of attacks required under the random attack scenario.

In this experiment, we count the average number of random requests sent by hackers that cause exceptions in the microservices. The experiment has been conducted 20 times and the averaged results are reported in Figure 7. One can see that all the strategies used by the MDSF force hackers to send more requests to succeed. Typically, the heterogeneity-preferred strategy, balanced strategy and randomness strategy make hackers have to send 82.9%, 46.7%, and 35.2% more requests to complete the attack than the baseline, respectively. This is because MDSF selects the replacement image with heterogeneity. Thus, attackers need more attempts, which demonstrates that MDSF can have better active defense power to defend against random attacks.

#### 4.3.2. Study of the Continuous Attack

**Figure 8:**
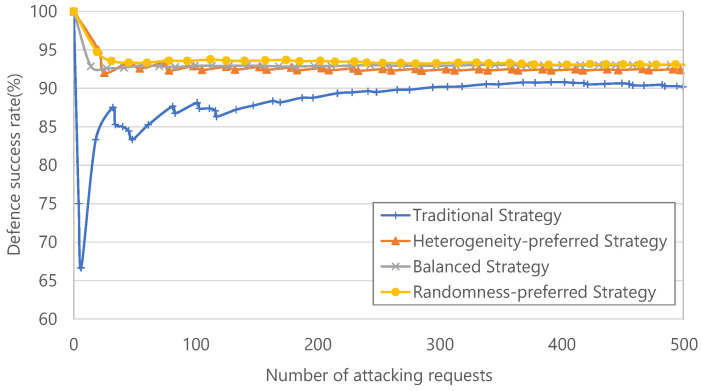
Defence success rate under the continuous attack scenario.

In this experiment, we let the hacker continuously send the same request 500 times to attack microservices with unknown vulnerabilities. We evaluated the performance of each strategy based on the defence success rate. The successful defence depends on whether all equivalent services can output the same response. As shown in Figure 8, one can see that all the strategies proposed by MDSF can achieve higher defence success rate than the traditional moving-target active defense framework. Typically, the randomness-preferred strategy reaches the best performance, as it can increase the defence success rate by 9.41%,9.18%,8.49%,8.43%,7.65%,7.43%, under 50,100,200,300,400,500 attacking requests, respectively, over the traditional strategy in the moving target framework. This is because the more randomness the mimic images selected in the SIG, the more difficulty the attackers had in finding the regular pattern and carrying out the continuous attacks. Thus, one can see that MDSF with randomness-preferred sequence can always perform a stable defence success rate against continuous attacks.

#### 4.3.3. Study of the System Performance

**Figure 9:**
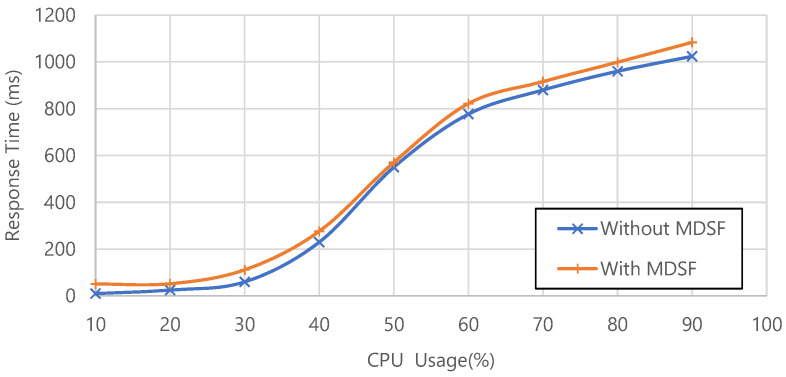
Microservice response time under different CPU usage.

To study the impact of MDSF on system performance, we analyze the response time of microservices deployed in a Kubernetes environment. During performance testing, we evaluate this response time by measuring the average time to complete the same API request from a single client. By adjusting the concurrency of requests, we record the average response time of microservices with and without MDSF under different CPU usage, as shown in the Figure 9. It can be seen that the response time of the microservice becomes longer as the CPU usage of the Kubernetes cluster increases. It also shows that services using MDSF always take longer response time than the original microservices without MDSF. The difference is about 30–70 ms, which is mainly due to the delay caused by mimic controller processing, which can be reduced in further research. Overall, the performance degradation brought from MDSF vulnerability protection is acceptable.

## 5. Limitation Discussion

From the above discussions, MDSF can defend against attacks that exploit unknown vulnerabilities. However, there are some limitations in protecting microservices from some specific attacks. First, MDSF cannot deal with denial-of-service attacks such as denial of service (DoS) and distributed denial of service (DDoS). This is because such attacks attempt to exhaust the target’s resources, making application services unavailable, rather than causing the target to generate abnormal output. Second, MDSF cannot protect microservices that produce inconsistent results (e.g., generate random results). Note that equivalent variants of such services can not guarantee the consistent output results for the same input, so MDSF cannot detect attacks by comparing the output results. Third, MDSF cannot defend against attacks that exploit inherent flaws in sensor networks (e.g., replay attacks, etc.). This is because all the equivalent variants have identical inherent defects, which will generate the same type of error outputs.

## 6. Conclusions

In this paper, we propose a novel system to strengthen container security using the mimic defense framework from unknown continues attack. Our proposed method can not only identify unknown vulnerabilities in images, but can also avoid continuous attack. By constructing the resource pool with image variant, the consistency of execution results from group of images can identify vulnerabilities in images. Moreover, we created a graph-based schedule strategy to maximize randomness and heterogeneity of the image group transformation. Finally, We use Kubernetes to realize the proposed system. Experimental results show that MDSF can make hackers have to send 54.9% more random requests to complete the attack and increase the defence success rate by around 8.16% compared to the traditional strategy to avoid the continuous unknown attacks. Further research directions include theoretical study on the optimal value of defense strategies to meet different levels of security requirements for microservices in the IoT field. Additionally, the theoretical defense efficiency of the mimic defense framework will be further explored.

## Figures and Tables

**Table 1:** Devices configuration settings.

Node	CPU	Memory	Disk	IP Address
Master	Intel Xeon E5 2687 2.70 GHz processor	256 G	1T	172.16.0.10
WorkerA	Hygon C86 7159 2.0 GHz 32-core Processor	256 G	2T	172.16.0.30
WorkerB	Hygon C86 7159 2.0 GHz 32-core Processor	256 G	2T	172.16.0.31
WorkerC	Hygon C86 7159 2.0 GHz 32-core Processor	256 G	2T	172.16.0.32

**Table 2:** Detail information of the mimic layers.

Layer	Candidate	Version	Vulnerability	Vulnerability Description	Size
1	CentOS	7.0	CVE-2020-5291	Can be used to gain root permissions	209 MB
	Ubuntu	14.04	CVE-2014-1424	Allow attackers to bypass AppArmor policies	197 MB
2	Apache	2.4	CVE-2017-7679	Can read byte past the end of the buffer	54 MB
	Nginx	1.10	CVE-2017-7529	Leak of information triggered by specially request	66 MB
	Lighttpd	1.4.11	CVE-2018-19052	Potential path traversal of a single directory	78 MB

**Table 3:** Mapping relationship between mimic images and mimic layers.

Serial Number	Image ID	Image Tag	Mimic Layer 1	Mimic Layer 2
0	1816f2528ad0	app:CentApch	CentOS	Apache
1	99cc511d5595	app:CentNgnx	CentOS	Nginx
2	0689b34165ff	app:CentLght	CentOS	Lighttpd
3	6fa35b2ba1c5	app:UbunApch	Ubuntu	Apache
4	60b82fc64a88	app:UbunNgnx	Ubuntu	Nginx
5	9a4486e0a7a0	app:UbunLght	Ubuntu	Lighttpd

**Table 4:** The image replacement sequence generated from the MDSF scheduling strategies.

	α	β	Sequences *S*
** $S_{0}$ **	-	-	G(0,1,2) ➀ → G(0,2,3) ➀ → G(2,3,5) ➁ → G(1,3,4) ➁ → G(0,2,4) ➀ → G(2,4,5) ➀ → G(2,3,4) ➀ → G(3,4,5) ➀ → G(1,4,5) ➀ → G(1,2,4) ➀ → G(1,2,3) ➂ → G(0,4,5) ➀ → G(3,4,5) ➀ → G(0,3,4) ➁ → G(0,2,5) ➁ → G(1,4,5) ➀ → G(0,1,4) ➂ → G(2,3,5) ➀ → G(1,2,5) ➁ → G(0,3,5)
** $S_{1}$ **	10	1	G(0,2,4) ➂ → G(1,3,5) ➂ → G(0,2,4) ➂ → G(1,3,5) ➂ → G(0,2,4) ➂ → G(1,3,5) ➂ → G(0,2,4) ➂ → G(1,3,5) ➂ → G(0,2,4) ➂ → G(1,3,5) ➂ → G(0,2,4) ➂ → G(1,3,5) ➂ → G(0,2,4) ➂ → G(1,3,5) ➂ → G(0,2,4) ➂ → G(1,3,5) ➂ → G(0,2,4) ➂ → G(1,3,5) ➂ → G(0,2,4) ➂ → G(1,3,5)
** $S_{2}$ **	1	1	G(0,1,2) ➁ → G(0,4,5) ➁ → G(2,3,5) ➁ → G(0,3,4) ➁ → G(1,2,3) ➂ → G(0,4,5) ➁ → G(1,3,5) ➁ → G(0,1,4) ➁ → G(0,2,3) ➁ → G(0,1,4) ➁ → G(0,2,3) ➁ → G(1,3,5) ➂ → G(0,2,4) ➁ → G(1,4,5) ➁ → G(1,2,3) ➁ → G(1,4,5) ➁ → G(0,1,2) ➁ → G(0,3,4) ➁ → G(2,3,5) ➁ → G(0,2,4)
** $S_{3}$ **	1	10	G(0,1,3) ➁ → G(2,3,5) ➁ → G(0,1,2) ➁ → G(2,4,5) ➁ → G(0,3,5) ➁ → G(1,2,3) ➁ → G(0,1,5) ➁ → G(0,2,3) ➁ → G(1,2,5) ➁ → G(0,2,4) ➁ → G(1,3,4) ➁ → G(0,4,5) ➁ → G(1,2,4) ➁ → G(1,3,5) ➁ → G(2,3,4) ➁ → G(0,1,4) ➁ → G(3,4,5) ➁ → G(0,2,5) ➁ → G(0,3,4) ➁ → G(1,4,5)

## Data Availability

Not applicable.
